# A Novel Biosorbent for Preconcentrations of Co(II) and Hg(II) in Real Samples

**DOI:** 10.1038/s41598-019-57401-y

**Published:** 2020-01-16

**Authors:** Sadin Ozdemir, Ersin Kılınç, Sen Fatih

**Affiliations:** 1 Food Processing Programme, Technical Science Vocational School, Mersin University, TR-33343 Yenisehir, Mersin Turkey; 2 Department of Chemistry and Chemical Processing Technologies, Vocational School of Technical Sciences, Dicle University, 21280 Diyarbakır, Turkey; 3 Sen Research Group, Department of Biochemistry, Faculty of Art and Science, Dumlupinar University, Kutahya, Turkey

**Keywords:** Pollution remediation, Biocatalysis

## Abstract

A new biosorbent, composed of Amberlite XAD-4 loaded with *Anoxybacillus kestanboliensis*, was developed and surface morphologies were investigated by SEM and FT-IR. It was used for solid phase column preconcentrations of Co(II) and Hg(II) before their measurements by ICP-OES. LODs were calculated as 0.04 and 0.06 ng mL^−1^ for Co(II) and Hg(II) respectively. The maximum biosorption capacities were determined as 24.3 and 27.8 mg g^−1^ for Co(II) and Hg(II) respectively. Preconcentration factors were achieved for Co(II) and Hg(II) as 80. The method validation was performed by analyzing certified reference materials. The new process was successfully utilized for the preconcentration of these metals in various food samples. It should be highlighted that the sensitivity of ICP-OES was critically improved by applying developed method. Hence, ICP-OES could be an effective alternative for ICP-MS and/or GF-AAS.

## Introduction

Some metal ions are known to be highly toxic. Therefore, a number of recent studies have focused on metal ion toxicity and carcinogenicity on human beings and other living organisms even at very low levels^1,2^. Among the various types of pollutants, toxic metal ions cannot be easily eliminated from nature^3^. Accumulation of toxic and deleterious metal ions in soft tissues of animals and humans even at low concentrations could be a major concern as they are not metabolized and can create significant damage to the body tissues^4^. Co and Hg are two typical metal ions in environmental samples which are also detected in plants and food samples. Toxicological influences such as vasodilation and cardiomyopathy are reported for Co exposure. Hg is also familiar as it is the most neurotoxic metal ion and can harm most of the human systems^3^.

The usage of the biological processes is an idea for the operation of environmental metal pollution. Biological processes for the bioremediation depend on the physical nature of binding sites and chemical conditions^4^. The utilization of biomass, including alive and dead microorganisms in the possibly retention and removal of metal ions from environmental samples, has acquired significant reliability over the past few years due to its efficiency, ecofriendliness and economic choice. The major properties of biosorbents are (a) having a variety of metal binding sites, (b) having small and identical sizes, (c) being less subject to interference from alkali-earth and alkali metals than ion-exchange resins (d) being generally cheaper than functionalized polymers (e) having the possibility to be obtained via green technologies^5^.

Concentrations of toxic metal ions are known as trace levels that are lower than the quantification limit of the instruments^6^. The determination, separation and analysis of low concentration of toxic metal ions in natural samples such as soil, food and water have become significant^7^. Therefore, it is necessary to develop analytical methods to preconcentrate and determine trace metal ions^6^.

Alternative methods are recommended for the preconcentration and separation of metals before their measurements. Solid phase extraction, coprecipitation, cloud point extraction, membrane filtration, electro deposition, liquid liquid extraction, photo-degradation, photocatalysis and, ion exchange are the methods for the preconcentration of metals in a variety of samples^8–13^. Solid phase extraction (SPE) has attained special interest related to features such as easy procedure, low operational time, high preconcentration factor, high retention, low cost, low pollution, flexibility and reusability of absorbents/biosorbents^14–16^.

The aim of the study is the development of an analytical method based on the use of thermophilic *Anoxybacillus kestanboliensis* immobilized biosorbent as a solid phase extractor for Co(II) and Hg(II). According to our literature survey, further researches are required for the use of immobilized thermophilic bacteria as a biosorbent material for the preconcentration of metal ions. Development of SPE sorbent based on immobilized thermophilic *A. kestanboliensis* on XAD-4 resin for the preconcentrations of Co(II) and Hg(II) is aimed.

## Materials and Method

### Instrumentation and chemicals

ICP-OES (PerkinElmer Optima™ 2100 DV, PerkinElmer,) was used for the measurements of Co(II) and Hg(II) concentrations at 228.616 and 194.168 nm, respectively. The ICP-OES operating conditions of were indicated in our previous paper (Delay time was set as 15 s extra to avoid the memory effect on Hg measurement and monitored routinly) pH was measured by Mettler Toledo MPC 227 0.1.0 cm × 10.0 cm filtration column was used for SPE experiments. Flow rate were controlled by using Watson-Marlow 323 peristaltic pump. FT-IR spectra was recorded on a Perkin-Elmer infrared spectrometer on KBr pellets. SEM images were obtained with a LEO 440 SEM, which was used for investigation of surface macrostructure.

1000 µg mL^−1^ of Co(II) and Hg(II) solutions were buyed from High Purity Standards, Charleston, SC, USA). Concentrated H_2_O_2_ (35%), HNO_3_ (65%), HCl (36.5–38.0%) and NH_4_OH were obtained from Sigma Aldrich, Germany. NCSZC 73014 tea leaves, NCS DC78301 river sediment and DORM2dogfish muscle were already available in the laboratory.

### Cultured of Thermophilic *Anoxybacillus kestanboliensis*

In this experimental study, *Anoxybacillus kestanboliensis* was isolated from Afyonkarahisar Omer spring mud sample, Turkey. The morphological and biochemical tests were analyzed. 16 S rRNA analysis was also made in OINTEK, ITU, Istanbul, Turkey for the identification of bacteria. Thermophilic *A. kestanboliensis* was cultured in 1 L glass flasks containing 0.25 L Nutrient Broth (NB) at 120 rpm and 55 °C for one day on a rotary shaker.

### Preparation of the Dried Dead *Anoxybacillus kestanboliensis* and loaded Biomass

The preparation of dried dead *A. kestanboliensis* and the loading of bacteria on XAD-4 was prepared according to Ozdemir, *et al*.^17^ and Ozdemir, *et al*.^18^ with some modification.

### General sorption studies

The model solution (50 mL), consisted of Cd(II), Ni(II), Co(II), Hg(II), As(III), Cu(II), Fe(II), Pb(II), Mn(II) and Cr(III) at 10.0 ng mL^−1^, was used for the SPE procedure. The solution pH was adjusted to 3.0 and 6.0, considering our previous experiences. They were passed through the SPE column at the flow rate of 1 mL min^−1^. Concentrations of metal ions were measured in eluate (5.0 mL of 1.0 mol L^−1^ HCl) by ICP-OES.

### Sample preparation

Soil and tap water (after flushing 1.0 min) samples were received from Mardin, Turkey. The others were bought from local market. 100 mL portions of aqueous samples were directly applied to the method. Food samples were firstly digested in an analytical microwave oven (Berghof MWS3). 1.0 g portion of samples were weighed. 5.0 mL of HNO_3_:HCl (1:1, v/v) was added, and then the mixture was heated on a hot plate. It was dried and 6.0 mL of HNO_3_:HCl:H_2_O_2_ (1:1:0.2, v/v/v) was added before being transferred into a microwave vessel. It was heated to 170 °C by microwave irradiation and kept for 5.0 min. Then, the temperature was heated to 200 °C in 15 min and the mixture was kept there for 1.0 min. Next, the temperature was reduced to 100 °C and the mixture was kept there for another 20 min. After these procedures, the last volume was completed to 50.0 mL and the solution pH was adjusted to the requested value prior to the SPE process. The portions of tea leaves (NCSZC 73014), river sediments (NCS DC78301) and dogfish muscles (DORM2) (0.5 g) were digested, applying the same procedure reported for the environmental and food samples.

## Results and Discussion

**Figure 1:**
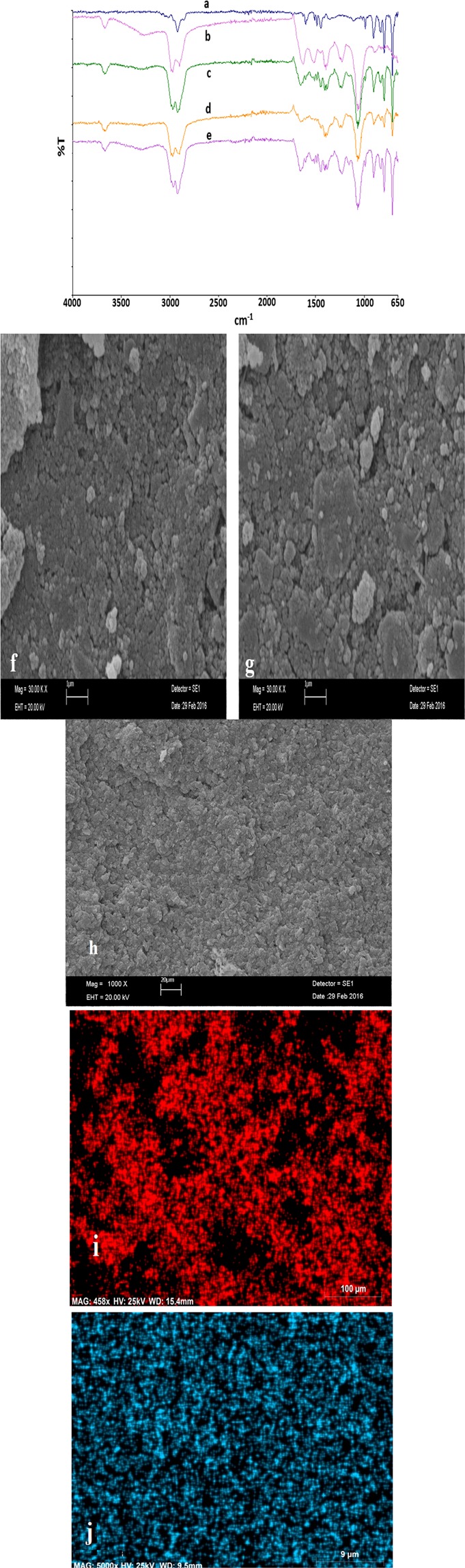
FT-IR spectral comparison of (**a)**. Amberlite XAD-4, (**b)**. *A. kestanboliens*, (**c)**. *A. kestanboliens* on Amberlite XAD-4, (**d)**. Co(II) loaded on *A. kestanboliens*-Amberlite XAD-4, (**e**). Hg(II) loaded on A*. kestanboliens*-Amberlite XAD-4, and SEM image of surface macrostructure of (**f)**. *A. kestanboliensis* immobilized XAD-4 with Co(II), (**g)**. *A. kestanboliensis* immobilized XAD-4 with Hg(II) (**h)**. *A. kestanboliensis* immobilized XAD-4 without Co(II) and Hg(II), (**i)**. EDX mapping of *A. kestanboliensis* immobilized XAD-4 with Co(II), (**j**). EDX mapping of *A. kestanboliensis* immobilized XAD-4 with Hg(II).

Surface functionalities of the *A. kestanboliensis* loaded XAD-4 resin with and without Co(II) and Hg(II) were examined through FT-IR spectral comparison. It was comparatively presented in Fig. 1. The peaks (from Fig. 1c) around 650, 1100, 1270, 1300 and 1400 cm^−1^ were attribuated to successfully loading of *A. kestanboliensis* to XAD-4. From Fig. 1b., the peaks on approximately 3750 cm^−1^, doublet on 2900 cm^−1^, 1700 cm^−1^, 1550 cm^−1^, 1450 cm^−1^ were attributed to O-H stretching of alcohol, N-H stretching from amine salt, C=O stretching, N-O stretching, C-H bending, respectively. By considering the hard and soft acids and bases theory, Hg and Co could be accepted as soft and borderline acids. Thus we can conclude that the targeted metal ions could interact with functional groups such as C_6_H_5_N, N_3_^−^, NO_2_^−^, SO_3_^−2^ and R^−^, C_2_H_4_, C_2_H_6_, RNC, CO, SCN^−^, R_3_P, R_2_S, RSH, RS^−^, S_2_O_3_^−2^ on bacteria loaded resin surface. Interactions could be due to complexation, ion exchange as well as physical adsorption^17^. No different peaks were observed after interaction of Co(II) and Hg(II). However, approximately 10 cm^−1^ shifting were observed and attributed to metal ions complexation with functional groups of bacterial surface. SEM analysis of thermophilic *A. kestanboliensis* immobilized XAD-4 was presented in Fig. 1f,g. As shown in Fig. 1f,g, the porous surface of the immobilized thermophilic *A. kestanboliensis* was clearly seen. It is well known that the porous surface is significant because of the high interaction between analytes and surface functional binding groups.

A mixture of model solution including Cd(II), Ni(II), Co(II), Hg(II), As(III), Cu(II), Fe(II), Pb(II), Mn(II) and Cr(III) at 10.0 ng mL^−1^ concentration was utilized to SPE procedure. Considering the ICP-OES results, it was decided to optimize the method for Co(II) and Hg(II) as recovery percentages were clearly lower for other cations.

### Effect of pH

**Figure 2:**
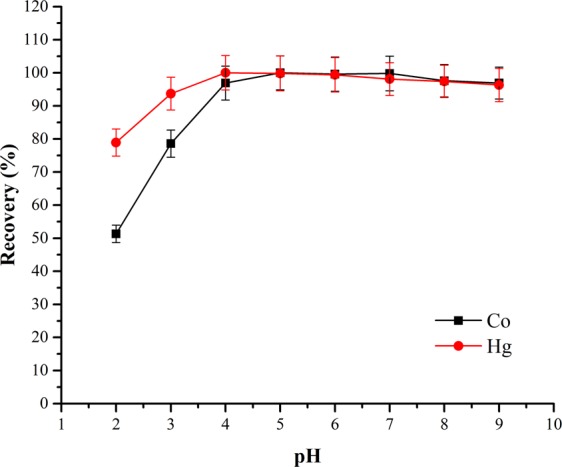
Effect of pH on the SPE preconcentrations of Co(II) and Hg(II).

pH solution influences the overall feature of the adsorbate and adsorbent^18^, and it also controls the electrostatic interaction between adsorbate and adsorbent^19^. The adsorbent chemical properties depend extremely on the solution pH^18^. The influence of pH on the recovery of Co(II) and Hg(II) of the immobilized thermophilic *A. kestanboliensis* on XAD-4 resin was investigated in the pH varieties of 2.0 to 9.0 (Fig. 2). The maximum pH for quantitative retentions of the tested metal ions by XAD-4 resin modified with thermophilic *A. kestanboliensis* was determined as 5.0 and 4.0 for Co(II) and Hg(II), respectively. It was determined that at lower pH, the existence of H^+^ complicates the cationic nature of Co(II) and Hg(II) metal ions which affects the biosorption rate as demonstrated in Fig. 2. At lower pH degrees, concentration of H^+^ is high and occupies the binding groups of cell surface and competes with analytes, as a result; the metal biosorption percentages decreased significantly^20^, however; at higher pH degrees, the metal ions displayed a trend to precipitate leading to lower biosorption^21^. All following experiments were tested at pH 5.0 and 4.0 for Co(II) and Hg(II), respectively.

### The effect of flow rate

The time course is significant for the biotechnological approach and controlling the performance of the biosorption process^5^. As represented in Fig. S1, flow rate also acts as a significant parameter in metal biosorption. For this reason, the determination of maximum flow rate is important to obtain the optimum metal ion retention. When the flow rate rised from 3.0 mL min^−1^ to 6.0 mL/min for recovery of Co(II) and Hg(II), the biosorption percentages reduced from 97.6% to 81.5% and 98.1% to 82.7% for Co(II) and Hg(II), respectively. The reason of the reduction in recoveries could be due to the insufficient contact time between binding sites of biomass and metal ions^22^. The maximum flow rate was determined as 2.0 mL min^−1^ for both studied metal ions. From this point of view, the flow rate of 2.0 mL min^−1^ was used in following studies.

### Effects of amounts of biosorbent and resin

The amount of biosorbents is also a critical parameter that affects the biosorption performance. Various concentrations of biosorbents can vary the quantity of the biosorbed metal ions on biosorbents as well as the recovery capacity^23^. The impact of the biosorbent concentration was examined in the variety of 100–300 mg. When the amount of biosorbent was increased from 50 mg to 200 mg, the recovery percentage was increased from 92% to 100%. This increase may be due to the availability of biosorbent metal binding sites as greater availability of specific biosorbents surfaces^24,25^. As seen in Fig. S2, the maximum biosorption yield was obtained at concentration of 200 mg of biosorbents. Appropriate quantity of biosorbents can bind the metal ions quantitatively from the metal solution samples. As the biosorbent concentration was less than 200 mg, the biosorbed metal ions reduced. On the other hand, in case of more than 200 mg of biosorbents, the biosorption percentage cannot change up to 300 mg. Similar findings reported by Sharma *et al*.^25^. Thus, following experiments were studied using 100 mg of biosorbents. In addition to this, the parameter of quantities of XAD-4 was experimented in the variety of 600–1000 mg. The results were given in Fig. S3. The retentions of Co(II) and Hg(II) rose up to 800 mg of XAD-4, however; that the amount was more than 800 mg of resin did not affect the metal ions retentions. So, 800 mg of XAD-4 was chosen as the best resin quantity for further processes.

### Influence of eluent type, concentration and volume

An acceptable eluent can effectively elute the biosorbed metal ions from surface binding sites for a high preconcentration factor. In addition to this, it should not influence the accurate determination of the metal ions and regeneration of solid phase extraction column^26^. For this purpose, various concentrations (0.5 mol L^−1^ and 1 mol L^−1^) and volumes (3 and 5 mL) of HCI and HNO_3_ were evaluated as an eluent. The experimental results of eluent types, concentrations and volumes were demonstrated in Table S1. When using 3 mL of 0.5 mol L^−1^ and 1 mol L^−1^ of HCI and HNO_3_, the recoveries were found as 90 ± 0.6% and 93 ± 0.8% and 88 ± 0.4% and 91 ± 0.9% for Co(II), respectively and 91 ± 0.8% and 94 ± 0.5% and 89 ± 0.7% and 92 ± 0.3% for Hg(II), respectively. The most satisfactory eluent was determined as 5 mL 1 mol L^−1^ HCI for the quantitative elution of Co(II) and Hg(II).

### Influence of matrix ions on retention of Co(II) and Hg(II)

The influences of some possible matrix ions such as K(I), Na(I), Mg(II), Ni(II), Zn(II), Ca(II), Cu(II), Fe(II), Cd(II) and Al(III), existing in natural samples on the uptakes of the Co(II) and Hg(II), were also tested by the addition, known as the amount of every matrix ion, into the sample solution containing the experimented metal ions. The tolerated quantities of every ion were less than 5% change on the absorbance. Not all the experimented matrix ions showed an interfering effect under experimental conditions (Table 1). These results demonstrated that the recommended SPE method for the examined metal ions should be applied to different samples.

**Table 1:** Effect of possible interfering ions on the SPE preconcentrations of Co(II) and Hg(II).

Recovery^a^ (%)			
Interferences	Interferic ion to metal ion ratio	Co(II)	Hg(II)
Na(I)	8000	98 ± 0.4	99 ± 0.2
K(I)	8000	97 ± 0.6	99 ± 0.7
Ca(II)	200	98 ± 1.4	100.1 ± 0.9
Mg(II)	200	98 ± 0.8	99 ± 0.5
Zn(II)	20	97 ± 1.4	98 ± 0.8
Fe(II)	20	97 ± 1.0	98 ± 0.9
Cd(II)	10	98 ± 1.3	99 ± 1.1
Cu(II)	10	97 ± 1.4	98 ± 0.7
Ni(II)	10	96 ± 0.9	97 ± 1.4
Al(III)	10	97 ± 0.7	97 ± 1.3

^a^Concentrations of the heavy metal ions are 10 µg L^−1^.

### Effect of sample volume and determination of enrichment factor

The parameter of the sample volume is very important to obtain a high preconcentration factor and credible analytical results and to determine the analytes at very low levels in natural samples^27^. As a result, the sample volume must be experimented in SPE studies. Various sample volumes were tested under the optimum experimental conditions for determination of the highest sample volume. The influence of the sample volume on the recoveries of the studied metal ions is represented in Fig. S4. The recovery yields of Co(II) and Hg(II) is higher than 95% up to sample volume of 400 mL. The preconcentration factor was found as 80 for both metal ions.

### Reusage of the SPE column

**Figure 3:**
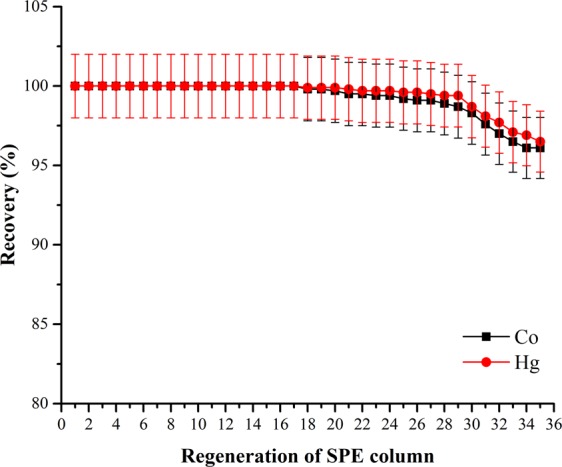
Effect of reusage of SPE column on the preconcentrations of Co(II) and Hg(II).

In SPE studies, the regeneration of the SPE column is also a significant factor for the economical perspective^17^.To analyze the regeneration of SPE column, 50 mL 1 mg L^−1^ of Co(II) and Hg(II) solutions were passed through the thermophilic *A. kestanboliensis* loaded onto XAD-4 column at a flow rate of 2.0 mL min^−1^. After 30 reusage of the SPE column, the recovery percentages of Co(II) and Hg(II) were found as 98.3% and 98.7%, respectively. It could be concluded that the developed column could be used up to 35 times with ≥95% retentions (Fig. 3).

### Analytical figures of merit

Analytical figures of merit were summarized in Table S2 in view of and preconcentration factor LOD, RSD, LOQ, linear range. Linear calibration curves for Co(II) and Hg(II) were acquired in the ranges of 0.25–12.5 ng mL^−1^ for Co(II) and Hg(II) with correlation coefficients as 0.9985 and 0.9989, respectively. LODs were 0.04 and 0.06 ng mL^−1^ for Co(II) and Hg(II), respectively. RSDs were found lower than 6.8%. Considering the 400 mL of original and 5 mL of last sample volume, the preconcentration factor was found as 80.

A list of the same analytical parameters for different procedures for Co(II) and Hg(II) preconcentrations is presented in Table 2^28–37^. It could be finalized that the developed procedure has more advantages due to its simplicity, being easy to handle and use of low cost bacterial biomass.

**Table 2:** Comparison of analytical characteristics of the preconcentrations methods for Co(II) and Hg(II).

Method	Instrument	LOD, ng mL^−1^		PF^1^		Linear range, ng mL^−1^		Ref.
		Co(II)	Hg(II)	Co(II)	Hg(II)	Co(II)	Hg(II)	
SPE on Amberlite XAD-2 resin anchored with pyrocatechol	FAAS	0.95	—	24	—	—	—	^28^
SPE ON 2-hydroxyacetophenone-functionalized polyurethane foam	FAAS	0.8	—	36	—	2.7–150	—	^29^
SPE on Duolite XAD 761 modified with a new Schiff base	FAAS	2.2	—	26	—	15–340	—	^30^
SPE on chelating resin	FAAS	0.44	—	150	—	5–900	—	^31^
Cationic micellar precipitation	ICP-OES	0.009	—	40	—	0.03–700	—	^32^
Magnetic SPE on magnetic core-shell nanoparticles modified with thiourea-derived chelating agents	Direct mercury analyzer	—	0.017	—	100	—	—	^33^
SPE on 2-nitroso-1-naphthol-4-sulfonic acid modified natural clinoptilolite zeolite	Derivative spectrophotometry	—	0.1	—	95	—	—	^34^
Cloud point extraction	ICP-OES	—	1.1	—	51.3	—	10–100	^35^
Magnetic SPE on Fe^3^O^4^@SiO^2^@PT nanocomposite	Cold vapor AAS	—	0.02	—	267	—	0.8–70	^36^
Preconcentration on an ion-imprinted polymer coated maghemite nanoparticles	FAAS	—	4.1	—	100	—	20–1000	^37^
SPE on Amberlite XAD-4 resin loaded with *Anoxybacillus kestanboliensis*	ICP-OES	0.04	0.06	80	80	0.25–12.5	0.25–12.5	This method

Tea leaves (NCSZC 73014), river sediment (NCS DC78301) and dogfish muscle (DORM2) were utilized to the developed procedure to validate the procedure. Results are presented in Table 3. As can be seen, good correlation was obtained between the determined and certified values.

**Table 3:** Langmuir and Freundlich isotherms for *Anoxybacillus kestanboliensis* loaded Amberlite XAD-4 as sorbent for Co(II) and Hg(II).

Metal ion	Langmuir isotherm			Freundlichisotherm		
	As (mg/g)	Kb (L/mg)	r^2^	K_F_	n	r^2^
Co	45.0	0.13	0.9954	6.0	1.86	0.9203
Hg	48.8	0.11	0.9975	5.6	1.72	0.9387

### Biosorption capacity

The biosorption capacity of the biomass is a major parameter as it detects how much biosorbent is necessary quantitatively^23^. 50.0 mL of 100.0 mg L^−1^ of Co(II) and Hg(II) solution at pH 5.0 and 4.0, respectively on 100.0 mg *A. kestanboliensis* loaded Amberlite XAD-4 were applied to the batch method. They were shaked at 120 rpm (2 g, radius of motor is 100 mm) for 120 min at 25 °C. Then the sorbent was separated at 10000 rpm (11200 g, radius of motor is 100 mm) for 10 min by centrifugation. The amounts of the Co(II) and Hg(II) in upper solution were directly determined. The pellet was digested in HNO_3_ before determination. The quantity of biosorbed Co(II) and Hg(II) was calculated using the equation given in literature^38^. The highest capacity of the thermophilic *A. kestanboliensis* loaded onto XAD-4 column was found to be 24.3 and 27.8 mg g^−1^ for Co(II) and Hg(II), respectively. The results demonstrated that the biosorption capacity of Co(II) and Hg(II) probably differ in size, level of hydration and their binding constant with the biosorbent^39^. In addition, results were utilized to Langmuir and Freundlich isotherms to define the mechanism of biosorption. Results are shown in Table 3. It was possible to conclude that biosorptions of Co(II) and Hg(II) on *A. kestanboliensis* loaded onto XAD-4 were mono-layer considering the high correlation coefficient of Langmuir isotherm.

### Real sample analysis

By considering the results from the validation experiments, it was decided to apply the developed method to real environmental and food samples for Co(II) and Hg(II) preconcentrations before their determinations by ICP-OES. In order to demonstrate the usability of the procedure, tap water, river water, mineral water, tuna fish, tomato, potato, macaroni, cabbage, red lentil, black and green teas, mint, baby rice powder and biscuit, coffee, bovine liver, gluten free biscuit, meet, beef and soil samples were analyzed (Table 4). Tap water, mint and baby biscuit samples were also spiked with known concentrations of analyte. They were recovered quantitatively. Hg(II) concentrations in tuna fish, potato and soil were determined after preconcentrations procedure, while it was lower than LOD for other samples. Concentrations of Co(II) in samples varied from <LOD to 16.9 ± 1.4 mg kg^−1^.

**Table 4:** Application of developed method for the preconcentrations of Co(II) and Hg(II).

Samples	Co mg kg^−1^	Hg mg kg^−1^
NCSZC 73014, certified	220 ± 20^a^	3.8 ± 0.8^a^
NCSZC 73014, determined	215 ± 26^a^	3.8 ± 0.6^a^
DORM2, certified	182 ± 31^a^	4.64 ± 0.26
DORM2, determined	179 ± 40^a^	4.58 ± 0.29
NCS DC78301, certified	16.5	0.22
NCS DC78301, determined	16.3 ± 1.1	±
Tap water	<LOD	<LOD
Tap water^b^	0.010 ± 0.001	0.009 ± 0.001
Tigris River water	<LOD	<LOD
Mineral water	<LOD	<LOD
Tuna fish	0.20 ± 0.02	0.026 ± 0.003
Tomato	0.66 ± 0.03	<LOD
Potato	0.65 ± 0.03	0.016 ± 0.001
Macaroni	0.044 ± 0.003	<LOD
Cabbage	0.66 ± 0.01	<LOD
Red lentil	0.14 ± 0.01	<LOD
Green tea	0.17 ± 0.01	<LOD
Black tea	1.6 ± 0.2	<LOD
Mint	0.051 ± 0.004	<LOD
Mint^b^	0.060 ± 0.003	0.009 ± 0.001
Baby biscuit	0.044 ± 0.002	<LOD
Baby biscuit^b^	0.053 ± 0.003	0.010 ± 0.001
Baby rice powder	0.069 ± 0.005	<LOD
Coffee	0.058 ± 0.004	<LOD
Gluten free biscuit	0.060 ± 0.005	<LOD
Bovine liver	0.86 ± 0.04	<LOD
Meat	0.083 ± 0.006	<LOD
Beef	0.069 ± 0.004	<LOD
Soil	16.9 ± 1.4	0.65 ± 0.03

^a^ng g^−1^.

^b^Spiked with known amounts of Co(II) and Hg(II) to give final concentrations as 0.010 mg kg^−^.

Direct determinations of Co(II) and Hg(II) at ultra-trace levels in environmental and food samples are not possible by ICP-OES. Thus, the use of GF-AAS and/or ICP-MS is an obligation for this purpose. We contribute to overcome this problem by developing Co(II) and Hg(II) specific SPE method. As a brief discussion of obtained results we can clearly highlight that the sensitivity of conventional ICP-OES was improved and the use of above mentioned instruments were eliminated. Results from certified reference samples sign the accuracy of the method. We can recommend the routine usability of the developed method.

## Conclusion

In this research paper, *A. kestanboliensis* was successfully loaded onto XAD-4, and used as SPE column material for the preconcentration and determinations of Co(II) and Hg(II). FT-IR and SEM analyzes were evaluated for surface characterization. The developed SPE column composed of bacteria loaded resin was used for over 35 cycles of biosorption/desorption without any loss in its biosorption behaviour. The developed method has much better features in comparison with literature due to high tolerance to foreign ions, too. Preconcentration factors were achieved as 80 for both of targeted metal ions. What we have achieved with this study is to critically increase the sensitivity of the ICP through the Co(II) and Hg(II). Hence low cost laboratories could use this environmentally friend method for routine analysis. The proposed method possesses lower LOD with a high preconcentration factor that makes it suitable for the preconcentrations of tested metals prior to their determinations by ICP-OES in real food samples.

## Supplementary information

A Novel Biosorbent for Preconcentrations of Co(II) and Hg(II) in Real Samples.
