# Supplementary material for: A Novel Biosorbent for Preconcentrations of Co(II) and Hg(II) in Real Samples
Source: Sci Rep. 2020 Jan 16;10:455. doi: 10.1038/s41598-019-57401-y (PMC6965309; doi:10.1038/s41598-019-57401-y)
Supplement: Supplementary file 1 — A Novel Biosorbent for Preconcentrations of Co(II) and Hg(II) in Real Samples. [file 41598_2019_57401_MOESM1_ESM.doc]

**SUPPORTING INFORMATION**

**A Novel Biosorbent for Preconcentrations of Co (II) and Hg (II) in Real Samples**

Sadin Ozdemir^a^, Ersin Kılınç^b*^, Fatih Sen^c*^

*^a^Food Processing Programme, Technical Science Vocational School, Mersin University, TR-33343 Yenisehir, Mersin, Turkey*

*^b^Department of Chemical and Chemical Processing Technologies, Vocational School of Technical Sciences, Dicle University, 21280, Diyarbakır, Turkey*

*^c^Sen Research Group, Department of Biochemistry, Faculty of Art and Science, Dumlupinar University, Turkey*

******Corresponding Author:****kilinersin@gmail.com,* [*fatihsen1980@gmail.com*](mailto:fatihsen1980@gmail.com)


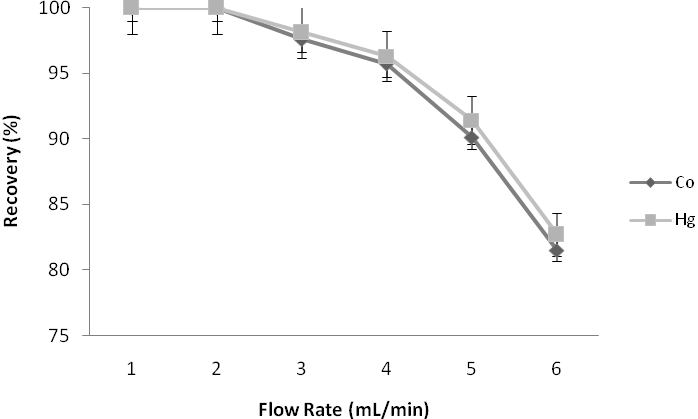


**Fig. S1.** Effect of flow rate on the SPE preconcentrations of Co(II) and Hg(II)


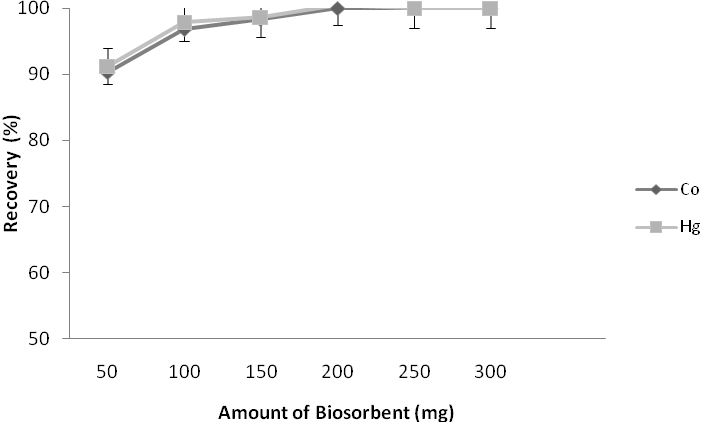


**Fig. S2.** Effect of amount of biosorbent on the SPE preconcentrations of Co(II) and Hg(II)


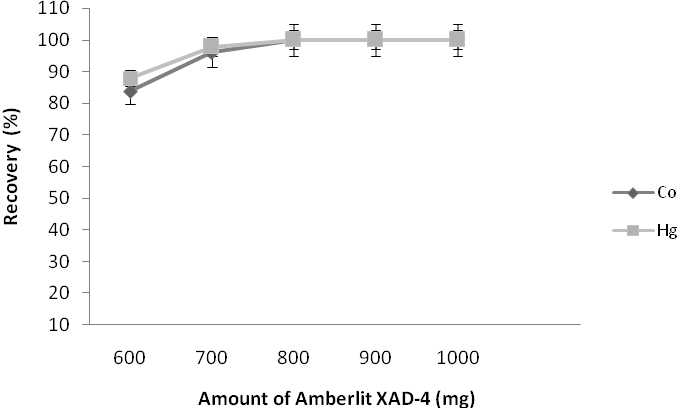


**Fig. S3.** Effect of amount of Amberlite XAD-4 on the SPE preconcentrations of Co(II) and Hg(II)

**Table S1.** Effect of elution conditions on the SPE preconcentrations of Co(II) andHg(II)

| **Eluent Type** | **Volume (mL)** | **Concentration (mol L^-1^)** | **Recovery^a^ (%)** | |
| --- | --- | --- | --- | --- |
| **HCI** |  |  | **Co(II) Hg(II)** | |
|  | 3 | 0.5 | 90±0.6 | 91±0.8 |
|  | 5 | 0.5 | 96±0.2 | 96±0.9 |
|  | 3 | 1 | 93±0.8 | 94±0.5 |
|  | 5 | 1 | 100.0±0.7 | 100.1±0.6 |
| **HNO_3_** | 3 | 0.5 | 88±0.4 | 89±0.7 |
|  | 5 | 0.5 | 93±1.3 | 95±0.6 |
|  | 3 | 1 | 91±0.9 | 92±0.3  98±0.9 |
|  | 5 | 1 | 97±1.2 |  |


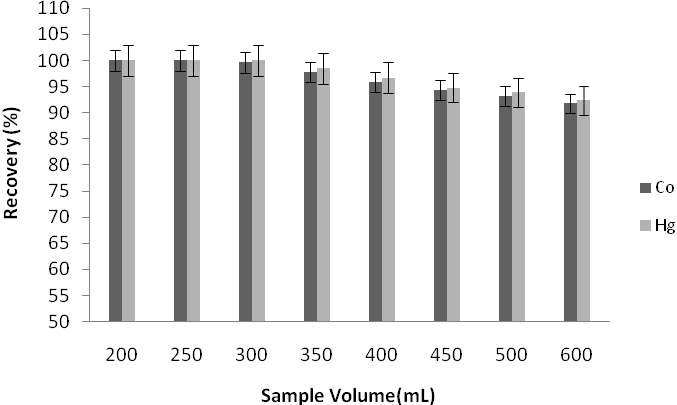


**Fig. S4.** Effect of sample volume on the SPE preconcentrations of Co(II) and Hg(II)

**Table S2.** Analytical characteristics of the developed method

| **Parameter** |  | **Co(II)** |  | **Hg(II)** |
| --- | --- | --- | --- | --- |
| LOD, ng mL^-1^ |  | 0.04 |  | 0.06 |
| LOQ, ng mL^-1^ |  | 0.13 |  | 0.22 |
| Linearrange, ng mL^-1^ |  | 0.25-12.5 |  | 0.25-12.5 |
| RSD^1^ |  | 6.8 |  | 4.9 |
| r^2^ |  | 0.9985 |  | 0.9989 |
| PF^2^ |  | 80 |  | 80 |

^1^Calculated for 1.0 ng mL^-1^ of Co(II) and Hg(II).

^2^Preconcentrationfactor
